# A Small RNA Isolation and Sequencing Protocol and Its Application to Assay CRISPR RNA Biogenesis in Bacteria

**DOI:** 10.21769/BioProtoc.2727

**Published:** 2018-02-20

**Authors:** Sukrit Silas, Nimit Jain, Michael Stadler, Becky Xu Hua Fu, Antonio Sánchez-Amat, Andrew Z. Fire, Joshua Arribere

**Affiliations:** 1 Department of Pathology, Stanford University, Stanford, CA, USA; 2 Department of Chemical and Systems Biology, Stanford University, Stanford, CA, USA; 3 Department of Bioengineering, Stanford University, Stanford, CA, USA; 4 Department of Molecular and Cell Biology, University of California, Berkeley, CA, USA; 5 Department of Genetics and Microbiology, Universidad de Murcia, Murcia, Spain; 6 Department of MCD Biology, University of California, Santa Cruz, CA, USA

**Keywords:** CRISPR, Small RNA, High throughput sequencing, Guide RNA, CRISPR RNA, crRNA processing, crRNA biogenesis, crRNA maturation

## Abstract

Next generation high-throughput sequencing has enabled sensitive and unambiguous analysis of RNA populations in cells. Here, we describe a method for isolation and strand-specific sequencing of small RNA pools from bacteria that can be multiplexed to accommodate multiple biological samples in a single experiment. Small RNAs are isolated by polyacrylamide gel electrophoresis and treated with T4 polynucleotide kinase. This allows for 3’ adapter ligation to CRISPR RNAs, which don’t have pre-existing 3’-OH ends. Pre-adenylated adapters are then ligated using T4 RNA ligase 1 in the absence of ATP and with a high concentration of polyethylene glycol (PEG). The 3’ capture step enables precise determination of the 3’ ends of diverse RNA molecules. Additionally, a random hexamer in the ligated adapter helps control for potential downstream amplification bias. Following reverse-transcription, the cDNA product is circularized and libraries are prepared by PCR. We show that the amplified library need not be visible by gel electrophoresis for efficient sequencing of the desired product. Using this method, we routinely prepare RNA sequencing libraries from minute amounts of purified small RNA. This protocol is tailored to assay for CRISPR RNA biogenesis in bacteria through sequencing of mature CRISPR RNAs, but can be used to sequence diverse classes of small RNAs. We also provide a fully worked example of our data processing pipeline, with instructions for running the provided scripts.

## Background

Genetic modules associated with Clustered Regularly Interspersed Short Palindromic Repeats (CRISPR) confer adaptive immunity in diverse prokaryotic hosts (Barrangou *et al*., 2007). Memories of invasive elements (such as viruses, plasmids, and other mobile elements) are stored interspersed between directed repeats of the CRISPR arrays in the host genome in the form of ‘spacers’ comprising the nucleic acid sequence of the molecular parasite (Brouns *et al*., 2008; Jackson *et al*., 2017). In order to identify subsequent infections by the same invader, the information contained in CRISPR spacers must be communicated to CRISPR-associated (Cas) endonucleases (Plagens *et al*., 2015). For the vast majority of CRISPR-Cas systems (phylogenetically grouped as ‘type I’ and ‘type III’ [Makarova *et al.*, 2011 and 2015]), this occurs through the activity of a family of CRISPR-associated endoribonucleases known as Cas6 (Charpentier *et al*., 2015; Hochstrasser and Doudna, 2015). The entire CRISPR array is transcribed as a precursor CRISPR RNA (pre-crRNA) molecule from the genome, and the Cas6 protein domain helps to process this transcript into a collection of mature CRISPR RNAs (crRNA) consisting of one CRISPR spacer each, flanked by portions of the CRISPR repeat sequence (Carte *et al*., 2008 and 2010; Haurwitz *et al.*, 2010). This mechanism is known as crRNA biogenesis. Cas6 endoribonucleases promote crRNA biogenesis through site-specific cleavage of the CRISPR repeat sequence, which generates 5’-OH and 2’3’-cyclic phosphate termini (Charpentier *et al*., 2015; Hochstrasser and Doudna, 2015). Site-specific cleavage at every CRISPR repeat results in the pre-crRNA molecule being chopped at regular intervals into almost equal-length crRNAs, each with a different spacer sequence (Charpentier *et al*., 2015; Hochstrasser and Doudna, 2015). Mature crRNAs are then loaded onto Cas effector complexes and serve as molecular guides that direct Cas enzymes to target DNA or RNA parasites based on sequence complementarity (Deveau *et al*., 2008; Marraffini and Sontheimer, 2008). The presence or absence of mature crRNAs isolated from bacterial cell populations can be used as a proxy for Cas6 activity. While biochemical methods have been developed to detect crRNAs (Carte *et al*., 2008 and 2010; Haurwitz *et al.*, 2010), high-throughput RNA sequencing can be used to assay for Cas6 activity unambiguously (Heidrich *et al*., 2015). Whole transcriptome sequencing is expensive and can be biased against specific classes of RNAs depending on the specific method of library preparation. Therefore, various small RNA sequencing protocols have been developed to preferentially detect mature crRNAs (Juranek *et al*., 2012; Richter *et al*., 2012; Heidrich *et al*., 2015).

Here, we present a multiplexed small RNA sequencing method to enable facile and reproducible comparisons of crRNA maturation between many different biological conditions at once, such as mutations in the Cas6 protein to assess the mechanism of Cas6 activity. This protocol builds on previous work on small RNA sequencing and ribosome profiling (Lau *et al*., 2001; Ingolia *et al.*, 2009; Guo *et al*., 2010; Kwon, 2011; Kivioja *et al.*, 2011). The assay features high sensitivity and dynamic range without expending a lot of sequencing bandwidth on other cellular RNAs, with the caveat that the full-length precursor transcript is not observed by small RNA sequencing.

## Materials and Reagents

1. Gel-Loading Pipette tips 0.5–200 μl (Thermo Fisher Scientific, Invitrogen™, catalog number: LC1001)
2. 0.6 ml microcentrifuge tubes (Sigma-Aldrich, catalog number: T5149)
3. Razor blade
4. Siliconized 1.5 ml microcentrifuge tubes (VWR, catalog number: 22179-004)
  *Manufacturer: BIO PLAS, catalog number: 4165SL*.
5. Plastic dish
6. Clear plastic film (Saran wrap, or equivalent)
7. Corning Costar Spin-X sterile 0.45 μm cellulose acetate centrifuge tube filters (Corning, catalog number: 8162)
8. 0.2 ml PCR tubes, MicroAmp (Thermo Fisher Scientific, Applied Biosystems™, catalog number: N8010540) or equivalent
9. Gel-Excision Pipette tips (Corning, Axygen^®^, catalog number: TGL-1165-R)
10. Heavy Phase Lock Gel in 2 ml tubes (Quantabio, catalog number: 2302830)
11. Corning tube top vacuum filtration system (Corning, catalog number: 430320)
12. Trizol reagent (Thermo Fisher Scietific, Invitrogen™, catalog number: 15596026)
13. Pre-Cast Novex 6% TBE-Urea polyacrylamide gels (Thermo Fisher Scientific, Invitrogen™, catalog number: EC6865BOX)
14. 10x TBE running buffer (Thermo Fisher Scientific, Invitrogen™, catalog number: AM9863)–dilute to 1x before use
15. GeneRuler Ultra Low Range DNA Ladder (Thermo Fisher Scientific, Thermo Scientific™, catalog number: SM1211)
16. 2x formamide gel loading dye (Thermo Fisher Scientific, Invitrogen™, catalog number: AM8546G)
17. SYBR Gold Nucleic Acid Gel Stain (Thermo Fisher Scientific, Invitrogen™, catalog number: S11494)
18. UltraPure glycogen (Thermo Fisher Scientific, Invitrogen™, catalog number: 10814010)
19. 200 Proof molecular biology grade ethanol (Sigma-Aldrich, catalog number: E7023)
20. UltraPure DNase/RNase-free distilled water (Thermo Fisher Scientific, catalog number: 10977035)
21. Polynucleotide Kinase (PNK) enzyme and buffer (New England Biolabs, catalog number: M0201S)
22. Ammonium acetate solution 7.5 M molecular biology grade (Sigma-Aldrich, catalog number: A2706)
23. 50% PEG 8000 (supplied with NEB T4 RNA ligase I)
24. Pre-adenylated 3’ adapter oligo: /5rApp/NNNNNNAGATCGGAAGAGCACACGTCT/3ddC/
25. T4 RNA ligase I (New England Biolabs, catalog number: M0204S)
26. NEB buffer 2 (New England Biolabs, catalog number: B7002S)
27. 5’ Deadenylase (New England Biolabs, catalog number: M0331S)
28. RecJ_f_ (New England Biolabs, catalog number: M0264S)
29. Acidified phenol:chloroform 1:1 mixture (Thermo Fisher Scientific, catalog number: AM9720)
30. Chloroform (Sigma-Aldrich, catalog number: 496189)
31. 5x First Strand Buffer (supplied with SuperScript II Reverse Transcriptase)
32. 0.1 M dithiothreitol (supplied with SuperScript II Reverse Transcriptase)
33. 10 mM dNTP mix (Thermo Fisher Scientific, Thermo Scientific™, catalog number: R0191)
34. SuperScript II Reverse Transcriptase (Thermo Fisher Scientific, Invitrogen™, catalog number: 18064014)
35. Reverse transcription primer:
  /5Phos/AGATCGGAAGAGCGTCGTGT/iSp18/CACTCA/iSp18/GTGACTGGAGTTCAGACGTGTGCTCTTCCGATCT
36. Pre-Cast Novex 10% TBE-Urea polyacrylamide gels (Thermo Fisher Scientific, Invitrogen™, catalog number: EC6875BOX)
37. 1 N sodium hydroxide solution (Merck, catalog number: SX0607H)
38. CircLigase ssDNA ligase and 10x reaction buffer (Lucigen, catalog number: CL4111K)
39. 1 mM ATP solution (supplied with circLigase)
40. UltraPure Agarose (Thermo Fisher Scientific, Invitrogen™, catalog number: 16500500)
41. 10x TAE (Thermo Fisher Scientific, catalog number: AM9869)–dilute to 1x before use
42. Ethidium bromide solution 10 mg/ml (Thermo Fisher Scientific, Thermo Scientific™, catalog number: 17898)
43. Phusion High-Fidelity PCR master mix (Thermo Fisher Scientific, Thermo Scientific™, catalog number: F531S)
44. Indexing primers:
  CAAGCAGAAGACGGCATACGAGATXXXXXXGTGACTGGAGTTCAGACGTGTGCTCTTCCG where the X_6_ barcodes correspond to Illumina TruSeq LT indexes AD001 to AD008 [ATCACG, CGATGT, TTAGGC, TGACCA, ACAGTG, GCCAAT, CAGATC, ACTTGA]
  Note: More indexing primers may be added as needed.
45. Universal PCR primer:
  AATGATACGGCGACCACCGAGATCTACACTCTTTCCCTACACGACGCTCTTCCGATCT
46. DNA gel loading dye 6x (Thermo Fisher Scientific, Thermo Scientific™, catalog number: R0611)
47. 25 bp DNA ladder
48. MinElute Gel extraction kit (QIAGEN, catalog number: 28604)
49. 5 M sodium chloride solution BioUltra for molecular biology (Sigma-Aldrich, catalog number: 71386)
50. 0.1 M EDTA solution, pH 7.5 (Merck, catalog number: EX0546A)
51. 1 M HEPES solution BioPerformance certified and 0.2 μm filtered (Sigma-Aldrich, catalog number: H3537)
52. 8 N potassium hydroxide solution (Sigma-Aldrich, catalog number: P4494)
53. 50 mM manganese chloride solution (supplied with circLigase)
54. Qubit dsDNA HS Assay Kit (Thermo Fisher Scientific, Invitrogen™, catalog number: Q32854)
55. 1 M Dithiothreitol solution BioUltra for molecular biology (Sigma-Aldrich, catalog number: 43816)
56. Glycerol for molecular biology (Sigma-Aldrich, catalog number: G5516)
57. 1 M magnesium chloride solution for molecular biology (Sigma-Aldrich, catalog number: M1028)
58. 20 mg/ml Acetylated bovine serum albumin (Thermo Fisher Scientific, catalog number: AM2614)
59. Polyacrylamide gel elution buffer (see Recipes)
60. 1 M HEPES/KOH buffer pH 8.3 (see Recipes)
61. 60% glycerol solution (see Recipes)
62. 5x adenylation buffer (see Recipes)

## Equipment

1. Scissors
2. XCell SureLock Mini-Cell (Thermo Fisher Scientific, Invitrogen™, model: XCell *Sure*Lock™ Mini-Cell, catalog number: EI0001)
3. Heated-lid thermocycler, Veriti 96-well (Thermo Fisher Scientific, Applied Biosystems™, model: Veriti™ 96-well, catalog number: 4375786) or equivalent
4. Transilluminator with 365 nm wavelength UV bulb (VWR, catalog number: 89131-464 or equivalent)
5. Tabletop microcentrifuge (Eppendorf, model: 5424 or equivalent, for use at room temperature and 4 °C)
6. Freezer capable of reaching −80 °C
7. Programmable water bath/heat block
8. Rotisserie tube rotator (VWR, catalog number: 10136-084 or equivalent)
9. 10 μl pipette
10. Gel electrophoresis power supply (Thermo Fisher Scientific, model: Owl™ EC1000XL or equivalent)
11. Owl EasyCast Mini Gel electrophoresis system (Thermo Fisher Scientific, Thermo Scientific™, model: Owl™ EasyCast™ B2) or equivalent
12. 100–1,000 μl pipetteh
13. Qubit 3.0 Fluorometer (Thermo Fisher Scientific, Invitrogen™, model: Qubit™ 3, catalog number: Q33216)

## Procedure

### Duration

**Figure 1:**
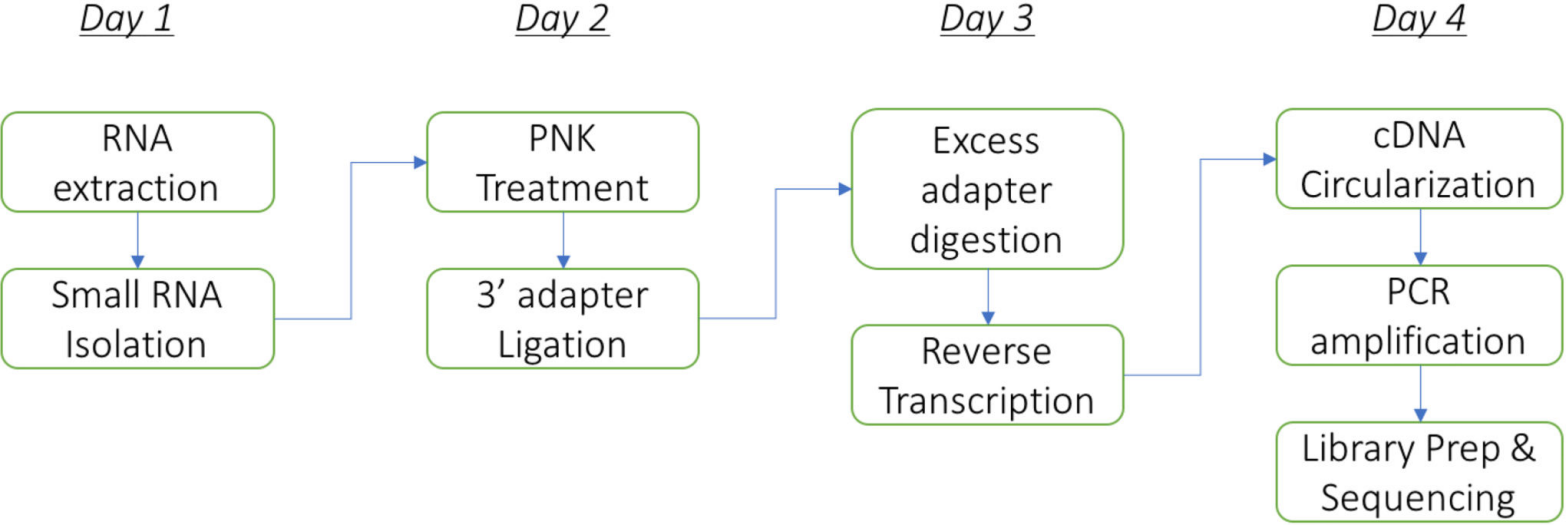
The flowchart of the major steps in the protocol

The protocol can be performed comfortably in 4 days (including RNA isolation from bacteria) as follows: Steps A1-A16 on day 1, A17-C5 on day 2, D1-E12 on day 3, and E13 onwards on day 4. The flowchart below summarizes the major steps in the protocol (Figure 1).

### RNA isolation from bacteria

RNA extraction methods will depend on the bacteria under study. The extraction method must avoid any column-based or size-dependent purification steps that could lead to preferential loss of small RNAs. We follow the manufacturer’s instructions provided with Trizol reagent for our model system *Marinomonas mediterranea*, a gamma-proteobacterium (like *E. coli*). We use no more than 200–500 μl of saturated *M. mediterranea* culture in Marine Broth 2216 for RNA isolation.

### Sequencing library preparation

**Figure 2:**
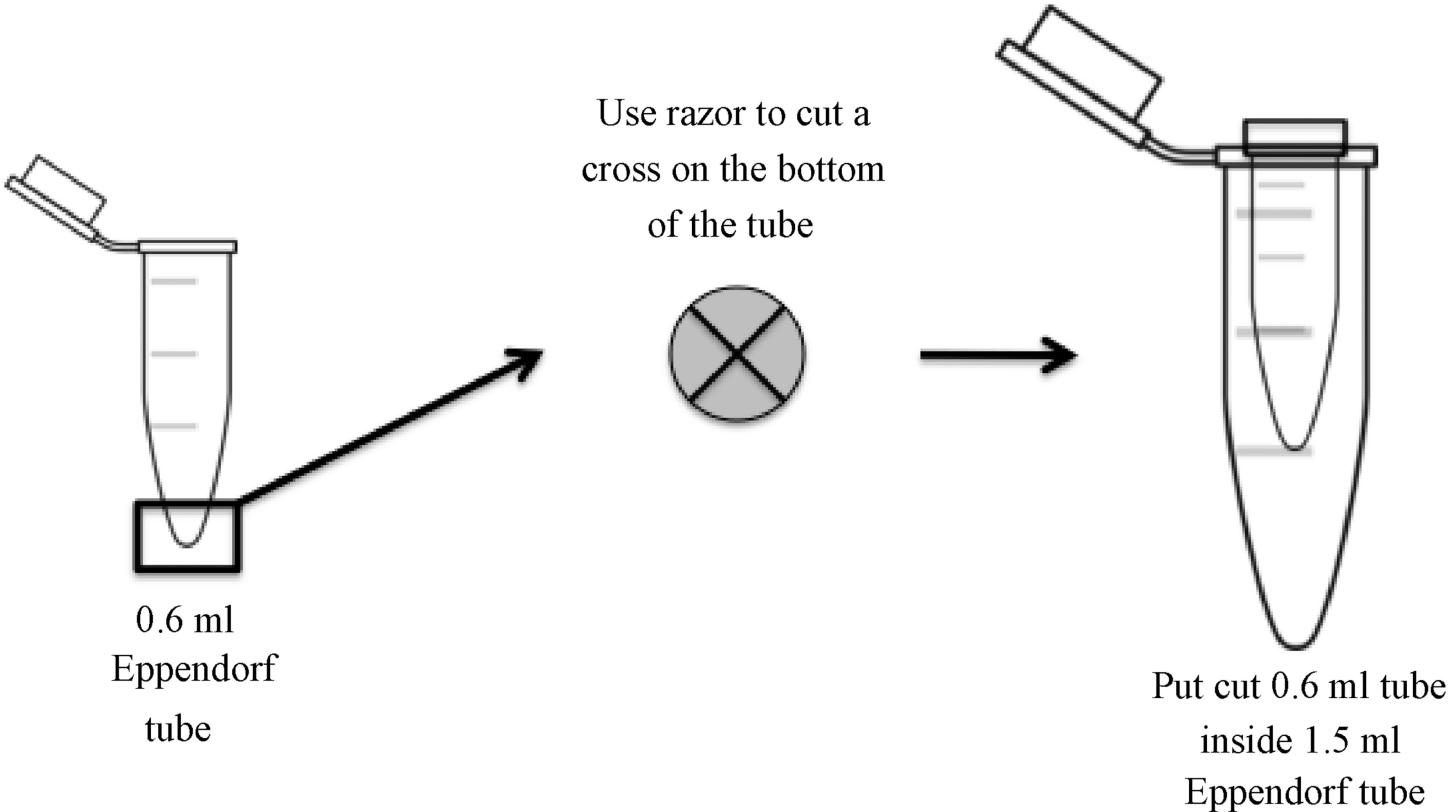
Longitudinal and transverse view of 0.6 ml tube for pulverizing polyacrylamide gel fragments A cross-shaped incision is made at the bottom of 0.6 ml centrifuge tube using a clean razor blade. The polyacrylamide gel fragment is pulverized as it is forced through the incision and into a 1.5 ml siliconized centrifuge tube by centrifugation.

**Figure 3:**
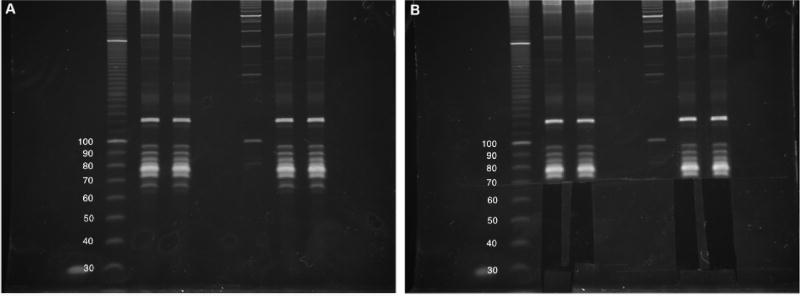
Small RNA size selection Four intact total RNA samples (6% denaturing TBE-Urea PAGE). Two biological replicates for each experiment were run side-by-side, with approximate DNA sizing ladders. Images cropped and brightness/contrast adjusted in Microsoft Word. B. Size selection of 25- to 75-nt RNAs, including lowest tRNA band. The Invitrogen 10 bp DNA ladder was used in this gel but has since been discontinued by the manufacturer.

**Figure 4:**
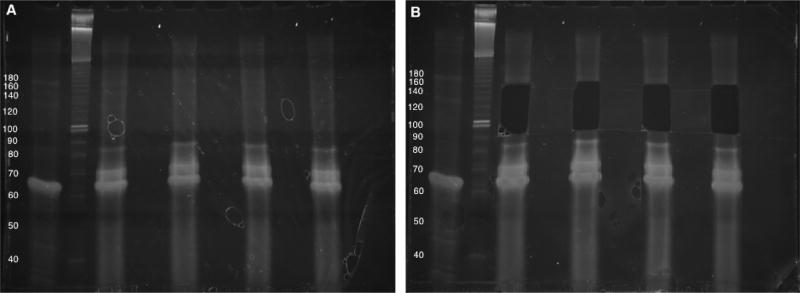
Purification of cDNA following reverse transcription Four cDNA sample (10% denaturing TBE-Urea PAGE). The first lane is the no-template control, followed by an approximate DNA sizing ladder. Brightness/contrast adjusted in Microsoft Word. B. Size selection of 100- to 160-nt cDNAs, avoiding bright high-molecular-weight bands. The Invitrogen 10 bp DNA ladder was used in this gel but has since been discontinued by the manufacturer.

**Figure 5:**
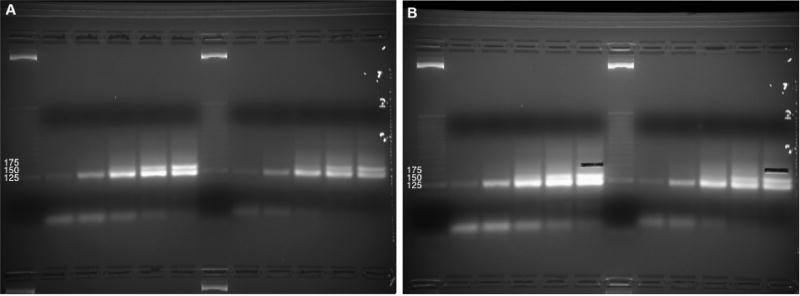
Library preparation by ‘blind’ gel excision PCR titrations of amplified sequencing libraries for two ccDNA samples (3–3.5% native agarose gel electrophoresis). 5 titrations for each ccDNA sample were run side-by-side along with a DNA sizing ladder. Brightness/contrast adjusted in Microsoft Word. B. Size selection of DNA at the 175 bp marker, above the bright band/doublet formed by amplification from empty ccDNA (*i.e*., without a small RNA insert). The Invitrogen 25 bp DNA ladder was used in this gel but has since been discontinued by the manufacturer.

- Small RNA isolation by denaturing polyacrylamide gel electrophoresis (PAGE)
  1. Assemble a pre-cast Novex 6% TBE-Urea denaturing polyacrylamide gel in the XCell SureLock Mini-Cell Electrophoresis System.
    Note: Remember to remove the gel comb and the green tape at the bottom of the gel cassette before assembling the electrophoresis cell.
  2. Fill the inside and outside chambers with 1x TBE running buffer, and pre-run the gel at 180 V for at least 30 min.
  3. Prepare samples of at least 5–10 μg total intact RNA and 0.1 μg Ultra Low Range DNA ladder in 2x formamide gel loading dye at a final concentration of 1x. We suggest keeping the total volume of each sample < 15 μl.
  4. Denature the samples and ladder by heating in a thermocycler with a pre-heated lid at 94 °C for 5 min, then immediately place in an ice-water slurry.
  5. While the samples are denaturing, thoroughly flush urea out of each gel well using a 100 μl pipette with the running buffer from the inner chamber several times.
  6. Load samples carefully with gel-loading pipette tips.
    Note: Leave 1–2 lanes between different RNA samples to reduce the amount of cross-contamination between experiments. We recommend including no more than 4–5 RNA samples (and one lane for the approximate sizing ladder) in a 10-lane gel.
  7. Run at 180 V until the bromophenol blue dye front (bottom band ~25 nt) reaches close to the end of the gel (about 35 min).
  8. While the gel is running, prepare gel elution tubes by making a small cross-shaped incision at the bottom of a 0.6 ml tube with a clean razor blade (see diagram for details) and placing it inside a 1.5 ml siliconized centrifuge tube. Do not remove the caps of either the 0.6 ml or 1.5 ml tubes (Figure 2).
    Note: Use of siliconized tubes is critical to avoid the loss of RNA due to non-specific binding to tube walls.
  9. Carefully disassemble the cassette and remove the gel. Stain with SYBR Gold diluted 1:5,000 in 1x TBE running buffer.
    Note: We typically use 3 μl of SYBR Gold in 15 ml running buffer and stain on a slowly rocking nutator in a small plastic dish for about 5 min at room temperature. Wear appropriate protective equipment to prevent exposure to SYBR Gold, and also to prevent contamination of samples with extraneous biological material.
  10. Transfer the gel onto a clear plastic film and place on a UV transilluminator (set at 365 nm wavelength).
  11. Carefully excise out gel fragments for each sample from the 25-nt marker upto the 75-nt marker, which should be just below a bright band corresponding to cellular tRNAs (Figure 3).
    Note: For a non-degraded RNA sample, there will most likely be no visible RNA in the excised gel fragment. We often include a small portion of the lowest visible tRNA band to serve as a carrier in subsequent steps. Intact tRNAs typically do not reverse transcribe efficiently and should not result in overwhelming contamination in the final dataset.
  12. Place each gel fragment in a separate elution tube.
  13. Centrifuge each elution tube at 20,000 *× g* at room temperature in a tabletop microcentrifuge for 1–3 min to force the gel fragment through the incision in the 0.6 ml tube and into the 1.5 ml siliconized tube. Carefully remove any leftover gel pieces in the 0.6 ml tube with a clean pipette tip, and place in the corresponding 1.5 ml siliconized tube. Discard the 0.6 ml tube.
  14. Add 300 μl of polyacrylamide gel elution buffer (see Recipes) into each 1.5 ml siliconized tube containing pulverized gel fragments, and vortex vigorously to make a uniform slurry.
  15. Place the tubes at −80 °C to freeze, then in a 37 °C water bath for 2 min to thaw. Vortex vigorously, and then repeat this step 2–3 times.
  16. Place the samples on ice for 1 min to cool, then incubate at 4 °C with shaking in a rotisserie tube rotator overnight to elute RNA from gel fragments.
  17. Centrifuge briefly to collect gel slurry at the bottom of the tube.
  18. Prepare filtration tubes by placing 0.45 μm sterile cellulose acetate filters in new 1.5 ml siliconized tubes.
  19. Widen the bore of 1,000 μl pipette tips using clean scissors, and transfer gel slurry to the filtration tubes.
  20. Collect RNA eluate by centrifugation at 16,000 *× g* for 2 min at room temperature, discard the filters, and add (in order) 1 μl (20 mg/ml) glycogen and 1 ml 100% ethanol to each sample.
  21. Precipitate nucleic acids by placing the tubes at −80 °C for 30 min.
  22. Centrifuge at 20,000 *× g* at 4 °C for 30 min, and discard the ethanol while taking care not to dislodge the pellet.
  23. Wash with 1 ml freshly prepared 70% ethanol, taking care to flush out the cap by inverting several times.
    Note: It is not necessary to vortex aggressively at this step. Vortexing can be helpful in dislodging the pellet, but excessive agitation, as well as use of more concentrated ethanol for washing will lead to pellet fragmentation and reduction in yield.
  24. Centrifuge at 20,000 *× g* for 2 min at room temperature to collect the pellet, pour off the ethanol and repeat the wash.
  25. Centrifuge briefly at room temperature to collect residual ethanol after the second wash step is complete. Remove remaining ethanol using a 10 μl pipette, taking care not to touch the pellet. Air dry for 3 min.
    Note: After removing residual ethanol with a pipette, 3 min is sufficient to dry the pellet. We typically dry under a flame to prevent dust from accidentally settling in the tubes.
  26. Resuspend pellet in 17 μl RNase-free water at room temperature.
    *Note: The added glycogen from Step A20 should result in a clearly visible pellet that may become translucent upon drying. The pellet will be easy to resuspend provided it has not been over-dried*.
- Polynucleotide kinase (PNK) treatment
  1. Denature RNA at 90 °C for 1 min in a heated-lid thermocycler, then plunge in ice for 1 min.
    Note: We use the entire RNA sample from the previous step, and do not attempt to measure its concentration since the amount of RNA is often below the detection limit of commercial assay kits.
  2. To 17 μl of the RNA sample, add (in order) 2 μl 10x PNK buffer and 1 μl PNK enzyme, and mix well by pipetting.
  3. Incubate at 37 °C for 1 h.
  4. Add (in order) 80 μl RNase-free water, 50 μl (7.5 M) ammonium acetate, and 500 μl 100% ethanol.
  5. Precipitate RNA as in Steps A21-A25.
  6. Resuspend pellet in 4.5 μl RNase-free water at room temperature.
    Note: The added glycogen from Step A20 should result in a clearly visible pellet that may become translucent upon drying. The pellet will be easy to resuspend provided it has not been over-dried.
- 3’ adapter ligation (without ATP)
  1. Pre-mix equal volumes of 5x adenylation buffer (see Recipes) and 50% PEG 8000 to make 4 μl mixture per sample (+ 20% extra to account for pipetting error).
  2. Transfer RNA samples to 0.2 ml PCR tubes, and add 4 μl of the mixture to each RNA sample. Mix well by pipetting.
    *Note: PEG 8000 is viscous and pre-mixing with 5x adenylation buffer helps to reduce viscosity and make dispensing to sample tubes easier. Mix by pipetting for as long as necessary until the solution appears uniform*.
  3. Heat sample to 98 °C for 1 min, plunge in ice for 1 min, then place at room temperature for the next step.
  4. Add (in order) 0.5 μl (100 μM) pre-adenylated 3’ adapter oligo, and 1 μl T4 RNA ligase I. Mix well by pipetting.
    Note: We keep the pre-adenylated 3’ adapter oligo at −80 °C and thaw on ice before use.
  5. Incubate in a thermocycler at 22 °C for 6 h. The reaction can be stored at 4 °C if performing this step overnight.
- Excess adapter digestion
  1. Pre-mix 78 μl RNase-free water and 10 μl NEB buffer 2 for each sample.
  2. Incubate RNA samples at 95 °C for 1 min, allow to cool and then add 88 μl of buffer mixture.
  3. Add 1 μl 5’ deadenylase, mix, and incubate at 30 °C in a thermocycler for 30 min.
  4. Add 1 μl RecJ_f_, mix, and incubate at 37 °C in a thermocycler for 30 min.
    Note: The 5’ deadenylase removes the /5rApp/ group from the free 5’ ends of un-ligated pre-adenylated adapters, thereby exposing the excess adapter molecules to digestion by the single-stranded-DNA-specific 5’ → 3’ exonuclease RecJ_f_.
  5. During the digestion step, pre-spin a heavy phaselock gel tube for each sample at 16,000 *× g* for 2 min at room temperature.
  6. Add 100 μl RNase-free water to each RNA sample, mix well, and transfer to a pre-spun phaselock tube.
  7. Add 200 μl acid-phenol:chloroform to each sample and mix by shaking vigorously by hand.
  8. Centrifuge at 16,000 *× g* at room temperature for 5 min.
  9. Add 200 μl chloroform to each sample in the same tube and mix gently by inversion.
  10. Centrifuge at 16,000 *× g* at room temperature for 5 min.
  11. Transfer the aqueous phase to a new 1.5 ml siliconized tube, and add (in order) 0.5 μl (20 mg/ml) glycogen, 100 μl (7.5 M) ammonium acetate and 1 ml 100% ethanol to each sample.
  12. Precipitate RNA as in Steps A21-A25.
  13. Resuspend pellet in 5.75 μl RNase-free water at room temperature.
    Note: The added glycogen from Step D11 should result in a clearly visible pellet that may become translucent upon drying. The pellet will be easy to resuspend provided it has not been over-dried.
- Reverse-transcription
  1. Prepare a reverse-transcription master mix, with 2 μl 5x First-strand buffer, 1 μl (0.1 M) dithiothreitol, and 0.5 μl (10 mM) dNTPs for each RNA sample (+ 20% extra to account for pipetting error).
  2. Transfer RNA samples to 0.2 ml PCR tubes, and add 0.25 μl (100 μM) reverse-transcription primer to each tube.
  3. Heat samples to 90 °C for 1 min, then plunge on ice for 1 min.
  4. Add 3.5 μl of reverse-transcription master mix to each sample.
    Note: Also maintain a ‘no-template’ control, which will allow for visualization of the reverse-transcription primer during the subsequent gel purification step.
  5. Add 0.5 μl SuperScript II reverse transcriptase to each reaction and mix well by pipetting.
  6. Incubate at 42 °C for 30 min in a heated-lid thermocycler to synthesize complementary DNA (cDNA).
  7. During this incubation step, set up and pre-run a Novex 10% TBE-Urea denaturing polyacrylamide gel in the XCell SureLock Mini-Cell Electrophoresis System at 180 V for at least 30 min as described in Steps A1-A2.
  8. Add 2 μl (1 N) sodium hydroxide to each reaction.
  9. Incubate at 70 °C for 15 min in a heated-lid thermocycler to hydrolyze RNA.
  10. Add 12 μl 2x formamide gel loading dye (*i.e*., at a final concentration of 1x) to each sample.
  11. Prepare 0.1 μg of Ultra Low Range DNA ladder in 2x formamide gel loading dye at a final concentration of 1x.
  12. Denature and run cDNA on pre-run gels as in Steps A4-A25, with the following modifications:
    - In Step A7, run the gel until the Xylene Cyanol dye front (top band ~55 nt) reaches close to the bottom of the gel (about 45–60 min).
    - In Step A11, excise gel fragments in the 100- to 160-nt range (processed CRISPR RNAs are generally in the ~50–100-nt range and the reverse transcription primer adds ~65-nt to the size of the desired small RNAs). Use the no-template control as a visual guide during gel excision, and avoid the bright bands formed in this lane (typically no higher than 90-nt) (Figure 4).
    - In step A16, cDNA elution should be carried out at room temperature.
  13. Resuspend the cDNA pellet in 17 μl water. Reserve half the sample and store at -20 °C as a backup.
- cDNA circularization
  1. Add 1 μl 10x circLigase reaction buffer, 0.5 μl (1 mM) ATP, and 0.5 μl (50 mM) manganese chloride solution to 8 μl cDNA in 0.2 ml PCR tubes.
  2. Add 0.5 μl circLigase enzyme. Mix well by pipetting.
  3. Incubate at 60 °C for 75 min in a heated-lid thermocycler.
  4. While the circularization reaction is proceeding, prepare enough 3–3.5% agarose gels (in 1x TAE with 0.5 μg/ml ethidium bromide) to accommodate 5 PCR lanes plus 1 DNA sizing ladder per cDNA sample.
    Note: We use the 1.5 mm thick 12-well combs supplied with Owl EasyCast B2 gel electrophoresis systems to make enough lanes for two cDNA samples.
  5. Stop the reaction by heating to 80 °C for 15 min. Use this circularized cDNA (ccDNA) sample directly as a template for PCR.
- PCR amplification and purification of sequencing libraries
  1. Prepare a PCR mix with 100 μl 2x Phusion Master Mix, 1 μl (100 μM) Universal PCR primer, and 100 μl water for each cDNA sample.
  2. Add 200 μl PCR mix to 5 μl ccDNA from Step F5. Add 1 μl of a different indexing primer for each sample.
  3. Split each reaction mixture into 5 separate 0.2 ml PCR tubes (40 μl each).
  4. Perform a PCR titration for each ccDNA sample by running each sub-reaction for a different number of cycles according to the following program:
    - 98 °C for 30 sec
    - N cycles of
      98 °C for 10 sec
      60 °C for 10 sec
      72 °C for 10 sec
    - hold at 10 °C
    Note: We typically perform titrations with N = 12, 15, 18, 21, and 24 cycles for each ccDNA sample.
  5. Add 8 μl of 6x DNA gel loading dye to each reaction.
  6. Load all 5 titrations for each sample side-by-side on the agarose gel. We suggest using the Ultra Low Range DNA ladder (~0.5 μg/lane) to demarcate sets of titrations of different ccDNA samples.
  7. Run the gel in agarose gel running buffer (1x TAE with 0.5 μg/ml ethidium bromide) at 3.6–3.7 V/cm for 1–2 h.
  8. Place the gels on a UV transilluminator (set at 365 nm wavelength). Choose the appropriate number of PCR cycles for each ccDNA sample by visually assessing the PCR titration (see Note below) and excise a gel slice containing the PCR amplicon corresponding to the size of the desired product using a 100–1,000 μl pipette fitted with gel excision tips. Expel each gel slice into a separate 1.5 ml centrifuge tube.
    *Note: A bright band corresponding to the ‘empty’ circularized ccDNA product (i.e., without a small RNA insert) should be visible in each lane. This may appear as a doublet as the number of PCR cycles (N) is increased. For most small RNA sequencing applications, the desired product will be ~50 bp above this bright band/doublet. For CRISPR RNA sequencing, we rarely ever see a visible smear at this size range, and cut ‘blindly’ using the DNA ladder and the location of the bright band/doublet (~125 bp) as a visual guide. We typically aim for the highest number of PCR cycles for each ccDNA sample while still safely avoiding the upward-smear from the bright ~125 bp band/doublet* (Figure 5).
  9. Extract DNA from the gel slices according to manufacturer’s instructions using the QIAGEN MinElute Gel Extraction kit.
  10. Quantify each purified DNA sample according to manufacturer’s instructions using the high-sensitivity double stranded DNA quantification kit accompanying the Qubit fluorometer.
  11. Calculate the approximate concentration of each sample according to the following formula:
    $$\frac{\text{sampleconcentration}(\frac{\text{ng}}{\mu l})}{660\frac{g}{\text{mol}}\times \text{averagelengthofDNAlibrary}(\text{bp})}\times 10^{6}=\text{concentration}(\text{nM})$$
  12. Pool the samples in equimolar amounts. The pooled library can be sequenced according to the specifications of your Illumina high-throughput sequencing services provider. We typically use the single-read configuration for 80 cycles for small RNA sequencing applications. For assessing pre-crRNA processing in *Marinomonas mediterranea*, we sequence no more than 1 million reads per sample, but this will depend on the level of expression of pre-crRNA in the species of interest.

## Data analysis

We include a worked example with sample data, which requires the following programs to be installed:

cutadapt (tested on v1.14; likely compatible with most other versions)
Python 2.7 (with numpy, matplotlib for plotting)

The usage formats of the provided python scripts are in ***bold italics***, followed by the specific commands in **bold** for the worked example with sample data. Start by downloading the worked example, and navigating to the worked_example/ directory in a unix terminal.

1. Demultiplex reads: Obtain the high-throughput sequencing data in ‘fastq’ format.
  - Sample and index reads will be in files *Undetermined_S0_L001_R1_001.fastq* and *Undetermined_S0_L001_I1_001.fastq* respectively, with the first read corresponding to the first index, the second read corresponding to the second index, and so on. A sample dataset is provided in the *sample_data* directory.
  - To segregate reads corresponding to each index, prepare a demultiplexing ‘key’–a tab separated text file with the first column containing the desired sample name, and the second containing the reverse complement of the corresponding TruSeq LT index (AD001-8). A sample file *deMultiplexKey_sample.dat* is provided.
  - Now run the *deMultiplexer.py* file as follows:
    ***python deMultiplexer.py <path_to_directory> <key>***
    ***e.g.,* python deMultiplexer.py sample_data/ deMultiplexKey_sample.dat**
    This generates a FASTQ file for each index provided in the key. Note how the files for samples 1–4 in the provided example are empty. The example dataset only contains reads corresponding to the index reads provided for samples 5–8.
    Move demultiplexed data (samples 5–8) to a separate directory
    **mkdir sample_demultiplexed**
    **cd sample_data/**
    mv sample[5–8]*.fastq ../sample_demultiplexed/
    **cd ../**
2. Trim adapters: The high-throughput sequencing data will contain Illumina adapter sequences. These are parts of the molecule that were necessary for sequencing on the Illumina flowcell.
3. Collapse reads to eliminate amplification bias: The assay design includes a random hexamer (NNNNNN) in the 3’ adapter sequence, which is ligated to every RNA molecule before reverse-transcription. This helps eliminate amplification bias in downstream steps and helps ensure that every read corresponds to a distinct RNA molecule in the biological sample.
  Both Steps 2 and 3 (trimming and collapsing) are performed in the provided example with the *dirRNAseqAnalyse.py* script (using the *readCollapser2.py* function, which must be in the same directory as the script) as follows:
  **python dirRNAseqAnalyse.py <path_to_directory> <maximum_read_length>**
  ***e.g.,* python dirRNAseqAnalyse.py sample_demultiplexed/ 80**
  The program produces a log file *dirRNAseqAnalyseLog.txt* which contains details of the adapter trimming step.
4. Convert to fasta: The *fastq2fasta.sh* script has been prepared anticipating the files that will be generated in Step 3 for sample data. Each line in this script processes one input fastq file to one output fasta file. Modify this file with your input files (with *.trimmed.collapsed.fastq* extensions) and output files (with *.fasta* extensions) as desired. Convert using the following commands:
  - Run the provided *fastqtofasta.sh* script:
    **sh fastq2fasta.sh**
    Move the trimming intermediates to a new directory:
    **mkdir sample_trimmed_collapsed**
    mv *.trimmed* sample_trimmed_collapsed/
  - Move the .fasta files to a new directory:
    mkdir sample_fasta
    mv *.fasta sample_fasta/
5. Filtering
  - Identify CRISPR derived reads: First, identify sequencing reads containing the 5’ end of the CRISPR direct repeat sequence. We require at least 5 contiguous bases in the sequencing read to match the first five bases of the CRISPR repeat. The CRISPR repeat of interest is supplied in the 1^st^ line of the parameters file *crRNAfigureMaker_params.txt*. Any sequence upstream of the start of the CRISPR repeat is removed.
  - Remove short matches: If the resulting processed repeat is shorter than 12 bases, also check to see if the 5 bases preceding the CRISPR repeat in the original read match one of the possible spacer endings from the CRISPR arrays in the bacterial genome. In this way, we require at least 12 bases from the CRISPR repeat, or 10 bases across the spacer-repeat junction for any read to qualify for downstream analysis. A dictionary of all possible native spacer endings from the type III-B CRISPR locus in the *Marinomonas mediterranea* MMB-1 genome is provided in *spacerEnds.dict*.
    *Note: The spacerEnds.dict file can be modified in any text editor, but its formatting must be preserved to prevent parsing errors in python*.
  - Assess match fidelity: If the read passes initial filtering, the processed repeat is then matched to the expected CRISPR repeat sequence. We require the repeat to be a left-anchored substring of the CRISPR repeat (*i.e*., the processed repeat may be shorter than the CRISPR repeat, but it must match at the 5’ end and cannot contain mismatches).
6. Measure levels of a reference gene: Next, count reads containing 25-nt substrings of a reference gene that is highly expressed and does not vary with the biological conditions under study. We use the isoleucine-tRNA sequence as a reference in *M. mediterranea* datasets, but this may need to be empirically determined based on your RNAseq data for your model. This sequence must be provided in the 2^nd^ line of the *crRNAfigureMaker_params.txt* file.
7. Plot a histogram of lengths of trimmed reads: Finally, plot a histogram of the lengths of the processed CRISPR repeats normalized to the reference gene. The 3^rd^ line of the *crRNAfigureMaker_params.txt* file is an arbitrary scaling parameter that controls the height of the Y axis in the plot. It can be changed to accommodate the levels of processed crRNAs relative to the reference gene in your dataset.
  Steps 5, 6, and 7 are performed by the *crRNAfigureMaker.py* script as follows:
  ***python crRNAfigureMaker.py <path_to_fasta_files> <keyword>***
  ***e.g*., python crRNAfigureMaker.py sample_fasta/ 8**

The *keyword* option specifies which files should be included in the analysis. The keyword can be any part of the file name. For instance, using the keyword ‘8’ will only process sample8.fasta in the worked example, while using the keyword ‘mpl’ will include all 4 sample files for processing, and using the keyword ‘sem’ will result in no files being included.

**Figure 6:**
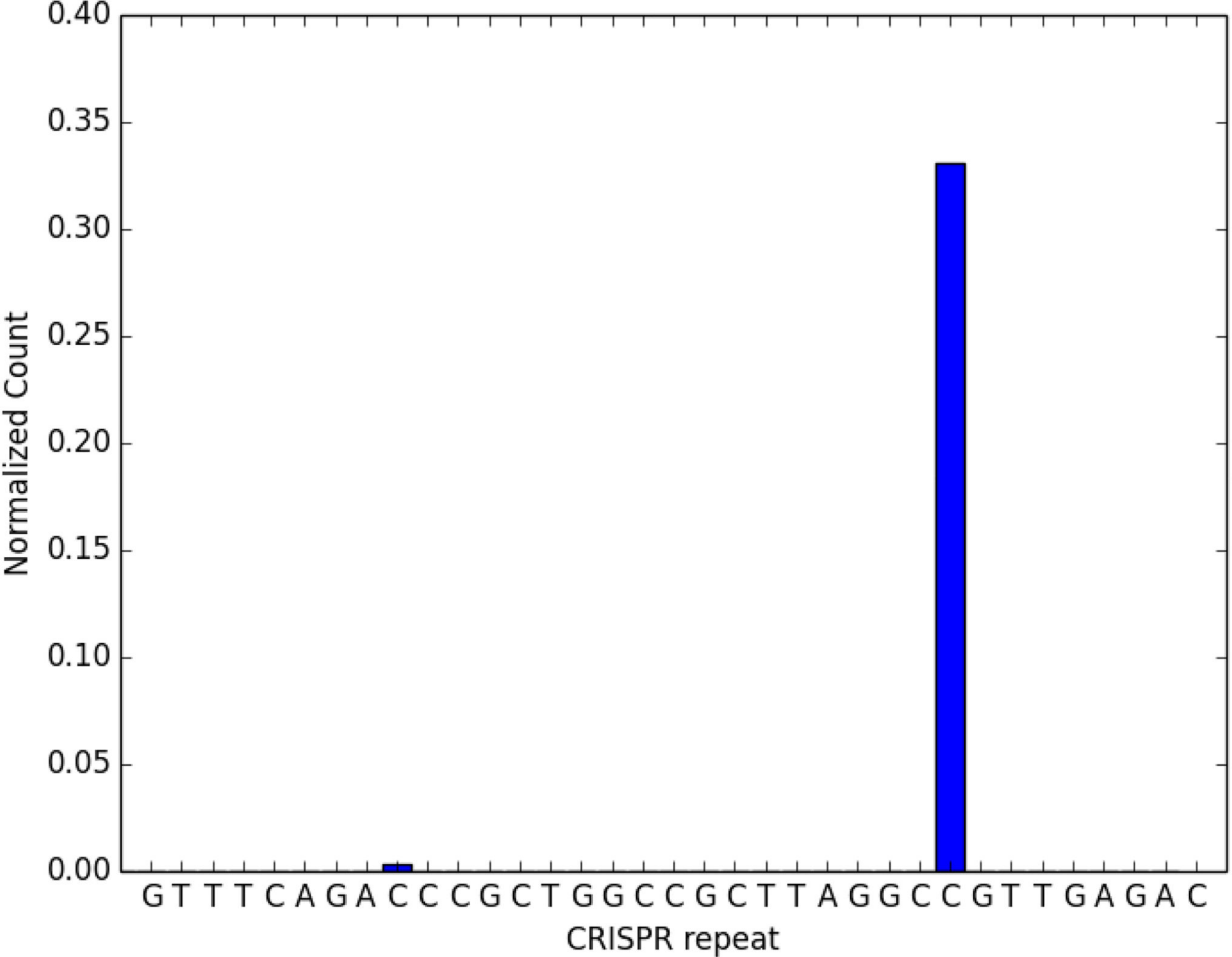
Expected output of code provided in the worked example Processed crRNA levels assayed by high throughput small RNA sequencing. This dataset has been artificially supplemented with sequences matching expected CRISPR-derived RNAs. The CRISPR repeat sequence from the 1^st^ line of the parameters file *crRNAfigureMaker_params.txt* is on the X-axis. The height of the bar at each base along the X-axis represents the relative proportion of crRNAs with 3’ ends at that base, normalized to the levels of the reference RNA (isoleucine tRNA; consistently the most abundant species encountered in our *M. mediterranea* datasets). The presence of a distinct 3’ end sequence in the population of CRISPR repeat containing RNAs indicates site-specific cleavage and processing of pre-crRNA.

The *crRNAfigureMaker_param.txt* file must be in the same directory as the code. Running the above command (*i.e.*, only processing sample8.fasta) should generate Figure 6 below.

The data files in the worked example are small subsets of our experimental data and have been artificially supplemented with sequences matching expected CRISPR-derived RNAs. Please refer to our public datasets at the NCBI Short Read Archive (SRP103952) to recreate the published graphs (Silas *et al*., 2017a). The following table specifies the accession numbers for the experiments that correspond to each of the relevant figure panels in (Silas *et al*., 2017a).

**Table:** 

SRA Dataset accessions	Figures
SRX2739032, SRX2739033, SRX2739034	4B, 4F
SRX2739035, SRX2739036, SRX2739037	4C, 4G
SRX2739026, SRX2739027, SRX2739028	4D, 4H
SRX2739029, SRX2739030, SRX2739031	4E, 4I

## Recipes

1. Polyacrylamide gel elution buffer
  300 mM NaCl
  1 mM EDTA
  *To make 50 ml:*

**Table:** 

3 ml	5 M sodium chloride solution
500	μl 0.1 M EDTA solution
46.5 ml	RNase free water
2. 1 M HEPES/KOH buffer pH 8.3
  Adjust the pH of 1 M HEPES solution with 8 N potassium hydroxide to 8.3
  Sterile filter using a 0.2 μm vacuum filtration unit
3. 60% glycerol solution
  To make 10 ml, mix 6 ml glycerol with 4 ml RNase-free water
  Sterilize by autoclaving
4. 5x adenylation buffer
  Note: Store in 1 ml aliquots at -20 °C up to 1 year.
  41% glycerol
  250 mM HEPES/KOH pH 8.3
  50 mM MgCl_2_
  16.5 mM DTT
  50 μg/ml Ac-BSA
  *To make 5 ml, mix:*

**Table:** 

3.4 ml	60% glycerol solution
1.25 ml	1 M HEPES/KOH buffer pH 8.3
250 μl	1 M magnesium chloride solution
82.5 μl	1 M dithiothreitol solution
12.5 μl	20 mg/ml acetylated BSA

## References

[1] Arribere JA, Cenik ES, Jain N, Hess GT, Lee CH, Bassik MC, Fire AZ (2016). Translation readthrough mitigation. Nature.

[2] Barrangou R, Fremaux C, Deveau H, Richards M, Boyaval P, Moineau S, Romero DA, Horvath P (2007). CRISPR provides acquired resistance against viruses in prokaryotes. Science.

[3] Brouns SJ, Jore MM, Lundgren M, Westra ER, Slijkhuis RJ, Snijders AP, Dickman MJ, Makarova KS, Koonin EV, van der Oost J (2008). Small CRISPR RNAs guide antiviral defense in prokaryotes. Science.

[4] Carte J, Pfister NT, Compton MM, Terns RM, Terns MP (2010). Binding and cleavage of CRISPR RNA by Cas6. RNA.

[5] Carte J, Wang R, Li H, Terns RM, Terns MP (2008). Cas6 is an endoribonuclease that generates guide RNAs for invader defense in prokaryotes. Genes Dev.

[6] Charpentier E, Richter H, van der Oost J, White MF (2015). Biogenesis pathways of RNA guides in archaeal and bacterial CRISPR-Cas adaptive immunity. FEMS Microbiol Rev.

[7] Deveau H, Barrangou R, Garneau JE, Labonte J, Fremaux C, Boyaval P, Romero DA, Horvath P, Moineau S (2008). Phage response to CRISPR-encoded resistance in *Streptococcus thermophilus*. J Bacteriol.

[8] Guo H, Ingolia NT, Weissman JS, Bartel DP (2010). Mammalian microRNAs predominantly act to decrease target mRNA levels. Nature.

[9] Haurwitz RE, Jinek M, Wiedenheft B, Zhou K, Doudna JA (2010). Sequence- and structure-specific RNA processing by a CRISPR endonuclease. Science.

[10] Heidrich N, Dugar G, Vogel J, Sharma CM (2015). Investigating CRISPR RNA biogenesis and function using RNA-seq. Methods Mol Biol.

[11] Hochstrasser ML, Doudna JA (2015). Cutting it close: CRISPR-associated endoribonuclease structure and function. Trends Biochem Sci.

[12] Ingolia NT, Ghaemmaghami S, Newman JR, Weissman JS (2009). Genome-wide analysis *in vivo* of translation with nucleotide resolution using ribosome profiling. Science.

[13] Jackson SA, McKenzie RE, Fagerlund RD, Kieper SN, Fineran PC, Brouns SJ (2017). CRISPR-Cas: Adapting to change. Science.

[14] Juranek S, Eban T, Altuvia Y, Brown M, Morozov P, Tuschl T, Margalit H (2012). A genome-wide view of the expression and processing patterns of *Thermus thermophilus* HB8 CRISPR RNAs. RNA.

[15] Kivioja T, Vaharautio A, Karlsson K, Bonke M, Enge M, Linnarsson S, Taipale J (2011). Counting absolute numbers of molecules using unique molecular identifiers. Nat Methods.

[16] Kwon YS (2011). Small RNA library preparation for next-generation sequencing by single ligation, extension and circularization technology. Biotechnol Lett.

[17] Lamm AT, Stadler MR, Zhang H, Gent JI, Fire AZ (2011). Multimodal RNA-seq using single-strand, double-strand, and CircLigase-based capture yields a refined and extended description of the *C. elegans* transcriptome. Genome Res.

[18] Lau NC, Lim LP, Weinstein EG, Bartel DP (2001). An abundant class of tiny RNAs with probable regulatory roles in *Caenorhabditis elegans*. Science.

[19] Makarova KS, Haft DH, Barrangou R, Brouns SJ, Charpentier E, Horvath P, Moineau S, Mojica FJ, Wolf YI, Yakunin AF, van der Oost J, Koonin EV (2011). Evolution and classification of the CRISPR-Cas systems. Nat Rev Microbiol.

[20] Makarova KS, Wolf YI, Alkhnbashi OS, Costa F, Shah SA, Saunders SJ, Barrangou R, Brouns SJ, Charpentier E, Haft DH, Horvath P, Moineau S, Mojica FJ, Terns RM, Terns MP, White MF, Yakunin AF, Garrett RA, van der Oost J, Backofen R, Koonin EV (2015). An updated evolutionary classification of CRISPR-Cas systems. Nat Rev Microbiol.

[21] Marraffini LA, Sontheimer EJ (2008). CRISPR interference limits horizontal gene transfer in staphylococci by targeting DNA. Science.

[22] Plagens A, Richter H, Charpentier E, Randau L (2015). DNA and RNA interference mechanisms by CRISPR-Cas surveillance complexes. FEMS Microbiol Rev.

[23] Richter H, Zoephel J, Schermuly J, Maticzka D, Backofen R, Randau L (2012). Characterization of CRISPR RNA processing in *Clostridium thermocellum* and *Methanococcus maripaludis*. Nucleic Acids Res.

[24] Silas S, Lucas-Elio P, Jackson SA, Aroca-Crevillen A, Hansen LL, Fineran PC, Fire AZ, Sanchez-Amat A (2017a). Type III CRISPR-Cas systems can provide redundancy to counteract viral escape from type I systems. Elife.

[25] Silas S, Makarova KS, Shmakov S, Paez-Espino D, Mohr G, Liu Y, Davison M, Roux S, Krishnamurthy SR, Fu BXH, Hansen LL, Wang D, Sullivan MB, Millard A, Clokie MR, Bhaya D, Lambowitz AM, Kyrpides NC, Koonin EV, Fire AZ (2017b). On the origin of reverse transcriptase-using CRISPR-Cas systems and their hyperdiverse, enigmatic spacer repertoires. MBio.

[26] Silas S, Mohr G, Sidote DJ, Markham LM, Sanchez-Amat A, Bhaya D, Lambowitz AM, Fire AZ (2016). Direct CRISPR spacer acquisition from RNA by a natural reverse transcriptase-Cas1 fusion protein. Science.

